# Focal Chromosomal Copy Number Aberrations Identify *CMTM8* and *GPR177* as New Candidate Driver Genes in Osteosarcoma

**DOI:** 10.1371/journal.pone.0115835

**Published:** 2014-12-31

**Authors:** Joeri Both, Oscar Krijgsman, Johannes Bras, Gerard R. Schaap, Frank Baas, Bauke Ylstra, Theo J. M. Hulsebos

**Affiliations:** 1 Department of Genome Analysis, Academic Medical Center, Amsterdam, The Netherlands; 2 Department of Pathology, VU University Medical Center, Amsterdam, the Netherlands; 3 Department of Pathology, Academic Medical Center, Amsterdam, The Netherlands; 4 Department of Orthopedic Surgery, Academic Medical Center, Amsterdam, The Netherlands; Johns Hopkins University, United States of America

## Abstract

Osteosarcoma is an aggressive bone tumor that preferentially develops in adolescents. The tumor is characterized by an abundance of genomic aberrations, which hampers the identification of the driver genes involved in osteosarcoma tumorigenesis. Our study aims to identify these genes by the investigation of focal copy number aberrations (CNAs, <3 Mb). For this purpose, we subjected 26 primary tumors of osteosarcoma patients to high-resolution single nucleotide polymorphism array analyses and identified 139 somatic focal CNAs. Of these, 72 had at least one gene located within or overlapping the focal CNA, with a total of 94 genes. For 84 of these genes, the expression status in 31 osteosarcoma samples was determined by expression microarray analysis. This enabled us to identify the genes of which the over- or underexpression was in more than 35% of cases in accordance to their copy number status (gain or loss). These candidate genes were subsequently validated in an independent set and furthermore corroborated as driver genes by verifying their role in other tumor types. We identified *CMTM8* as a new candidate tumor suppressor gene and *GPR177* as a new candidate oncogene in osteosarcoma. In osteosarcoma, CMTM8 has been shown to suppress EGFR signaling. In other tumor types, CMTM8 is known to suppress the activity of the oncogenic protein c-Met and GPR177 is known as an overexpressed upstream regulator of the Wnt-pathway. Further studies are needed to determine whether these proteins also exert the latter functions in osteosarcoma tumorigenesis.

## Introduction

Osteosarcoma is the most frequent primary bone tumor in children and adults [1]. The metaphyseal regions of long bones, i.e. the regions with high osteoblastic activity, which include the distal femur, proximal tibia and proximal humerus, are the main sites of primary tumor [2]. Tumor cells are thought to be of mesenchymal lineage and poorly differentiated, however they still produce osteoid [3]. With the introduction of chemotherapy the survival rate has risen to 60–75% in the last three decades of the 20^th^ century provided that no metastases are present at the time of diagnosis [4]. Survival rates decrease to less than 30% in metastatic disease [5], with the lungs as the primary site of metastasis [6].

Osteosarcomas have a complex karyotype that contain numerous chromosomal aberrations, which consist of gains, amplifications, deletions, translocations and overall aneuploidy [7]. Frequent copy number gains, suggestive for the presence of oncogenes, have been reported for chromosome regions 1p, 1q, 6p, 8q, and 17p11.2-p12 and copy number losses, suggestive for the presence of tumor suppressor genes, for chromosome regions 3q, 6q, 9, 10, 13, 17p13, and 18q. A number of oncogenes, including *MYC* and *RUNX2*, and tumor suppressor genes, notably *DOCK5*, *CCNE1*, and *LSAMP*, have been shown to be present in the affected regions, but other genes remain to be identified (see recent reviews by Martin *et al*. [8] and Kuijjer *et al*. [9]).

Each tumor genome harbours a mixture of genetic aberrations affecting genes that are directly responsible for its development (drivers) and random events whereby the affected genes have no biological significance (passengers) [10]. Distinguishing driver from passenger events in the cancer genome is crucial for our understanding of tumor development and will aid to identify novel oncogenes or tumor suppressor genes as potential targets for therapeutical intervention. The identification of driver genes has been a major challenge in earlier copy number studies due to the low resolution and consequently large sizes of the detected aberrations. The increase in resolution of array CGH and SNP platforms in recent years has allowed for the identification of small aberrations that went previously undetected. These sub-microscopic gains and losses, termed focal copy number aberrations (focal CNAs), were reported for different tumor types such as lung, breast, and colon cancer [11], [12]. Alike large CNAs of somatic origins, they are thought to be the result of a Darwinian-like, yet somatic, evolutionary selection process. Hence, a single gene in a focal CNA would give the tumor a selective growth advantage, which concept is instrumental in the fore laying study. The definition of focal CNAs does not have a biological basis. Based on pragmatic considerations, such as a limited number of genes, size definitions in previous studies and the size of CNVs as defined by Feuk et al [13] led us to select 3 Mb as the upper size limit for a focal CNA [14]. To our knowledge, only a few studies in primary human osteosarcoma that use high-resolution arrays have been published. Kuijjer *et al* (2012) [15] performed an integrative analysis of copy number and expression profiling in 29 osteosarcoma samples. They identified 31 candidate driver genes, mainly located in regions with recurrent chromosomal losses, of which a substantial number proved to be involved in genomic instability. Previously, we employed a comparable methodology to search for driver genes in chromosome region 17p11.2-p12 in osteosarcoma samples using high-resolution, genome-wide SNP array and expression microarray analyses [16]. In the present study, we combined both analytical tools to identify novel driver genes in chromosomal regions other than 17p11.2-p12. This was accomplished by identifying the recurrent focal CNAs in the genome of osteosarcomas and by gene expression analysis of the genes they affected.

## Materials and Methods

### Ethics statement

Clinical samples were irreversibly anonymised and results of scientific research could not be linked to individual patients. The Committee Medical Ethics of the Academic Medical Center (AMC) specifically waived approval for this study because it falls under paragraph 7∶467 Civil Law Code of The Netherlands.

### Patient samples

A total of 37 osteosarcomas, collected in the AMC, were analyzed in this study. The clinical data of the patients are summarized in Table 1. Sections of the tumors were H&E stained and reviewed by an experienced pathologist (J.Bras) to ensure high tumor cell content (>90%). Primary human fetal osteoblasts were cultured in osteoblast basal medium with osteoblast growth supplement (Cell Applications, Inc, San Diego, CA USA).

**Table 1:** Clinicopathological characteristics of patients and tumors.

	PatientCharacteristics	Number of samples (%)
Age	Mean	16.8
	Median	16
	Range	6–58
		
Sex	Male	20 (54.1)
	Female	17 (45.9)
		
Location of Primary Tumor	Femur	17 (45.9)
	Tibia/Fibula	9 (24.3)
	Humerus	5 (13.5)
	Axialskeleton	2 (5.4)
	Other	4 (10.8)
		
Histological subtype	Osteoblastic	31 (83.8)
	Fibroblastic	3 (8.1)
	Telangiectatic	2 (5.4)
	Unknown	1 (2.7)
		
Metastasis at diagnosis	No	34 (91.9)
	Yes	3 (8.1)

### Experimental design

DNA and RNA were isolated from alternating sections (10 µm) of fresh frozen tumor samples. A standard proteinase K digestion, followed by a chloroform extraction was used to obtain DNA of high molecular weight. Isolation of RNA was performed using Trizol (Invitrogen, Carlsbad, CA, USA) and a subsequent automated RNeasy protocol (QIAgen, Hilden, Germany). The quality of the RNA was assessed on a BioAnalyzer (Agilent, Santa Clara, CA, USA). Only samples with a RNA Integrity Number (RIN) score higher than 7.5 were included. Human primary fetal osteoblasts were cultured to a confluency of 90% and RNA was isolated to act as a reference in the microarray expression analyses.

### Single nucleotide polymorphism (SNP) and expression arrays

DNA of 26 osteosarcomas was hybridized for whole-genome copy number variation using Illumina HumanCNV370-Quad BeadChips as previously reported in Both *et al*. (2012) [16]. The array contained probes for 373,397 SNPs. Processing of DNA samples, hybridization, staining, and scanning of the BeadChips, and primary data extraction were all performed according to the Illumina Infinium II protocol at the array facility of ServiceXS (Leiden, the Netherlands).

Gene expression analysis using Illumina HumanHT-12 v3 Expression Beadchips (Illumina, San Diego USA) was performed on RNA of 31 osteosarcoma samples (of which 20 samples were also analyzed on the SNP array) as previously reported in Both *et al*. (2012) [16]. Each array contained 48,804 probes, spanning the human transcriptome. Labeling of RNA samples, hybridization, staining, and scanning of the Beadchips, and primary data extraction were all performed according to the Illumina Infinium II protocol, and under ISO 17025 certification, at the array facility of ServiceXS (Leiden, the Netherlands). Expression fold changes of a probe were determined by normalizing the intensity of the average signal in the tumor sample against the average signal in the primary human osteoblasts sample.

The SNP data and expression microarray data have been deposited in NCBI's GEO Omnibus and are accessible through GEO Series accession number GSE32964.

### SNP and expression array data analysis

Median normalized Log2ratios were segmented with Circular Binary Segmentation (CBS) [17]. After segmentation samples were mode normalized [18]. Chromosomal copy number changes were defined using the RpackageCGHcall [18]. Several criteria were applied to distinguish the tumor acquired and thus somatic copy number aberrations (CNAs) from the inherited and thus germline copy number variations, as reviewed by Feuk et al [13]. Germline copy number variants (CNVs) were removed from the dataset in 2 steps: 1) all DNA copy number changes of 3 Mb and smaller were removed if overlapping CNVs regions as archived in the database of genomic variants (DGV), population size and datasets can be found at: http://dgv.tcag.ca/ [19], 2) all copy number changes of 3 Mb and smaller that showed both recurrent (≥2) gains and losses were marked as CNVs and removed.

For each recurrent focal CNA, the smallest genomic overlap of the focal aberrations and the frequency in the dataset was determined and defined as high frequency region (HFR). Genes were retrieved using biomaRt (R/Bioconductor) and Ensembl (hg18/NCBI 36, ensemble 54).

Expression intensities were transformed to log2ratios with average primary osteoblast intensities as a reference. Array normalization was performed on the log2ratios using LOESS correction on each sample and a quantile normalization to correct for bias between the arrays [20].

For validation purposes the publicly available expression array of 84 osteosarcoma samples and 3 osteoblast samples previously published by Kuijjer et al. (2012) [15] was downloaded from NCBI's GEO Omnibus database (accession number GSE33383). This was normalized and analyzed using the same tools and settings as for our own dataset. All analyses were performed and plots were made using the statistical programming language R version 2.11.1 and Bioconductor packages (http://www.r-project.org).

### Selection bias

The 26 samples used for copy number profiling in this study were previously selected for the presence of an amplification event on chromosome arm 17p [16]. To determine whether this selection biased the type and frequency CNAs in our sample set, we compared the frequency of aberrations in our sample set with that of an unselected sample set. For the latter, the copy number data (raw log2ratios) of 20 samples, deposited by Kresse et al [21], was downloaded from http://www.ebi.ac.uk/arrayexpress/; file E-MEXP-1219 and processed with similar settings as for our samples. Matching of the platforms was performed as described by Van Wieringen *et al*. [22] with the “overlapPlus” function. Statistical differences between the matched series were determined using a Wilcoxon test with ties using the R-package CGHtest [18]. Apart from the expected higher frequency of 17p gain only one other chromosomal difference, a lower frequency of 7p gain, was observed (FDR q<0.05). No other significant differences were observed in the frequency of gains and losses between the two sets.

### Enrichment analysis

To test whether the focal aberrations were enriched for cancer related genes as published in the Cancer Census list (http://www.sanger.ac.uk/genetics/CGP/Census/) [23], enrichment analysis was performed as described previously [24].

## Results

### Focal aberrations

**Figure 1:**
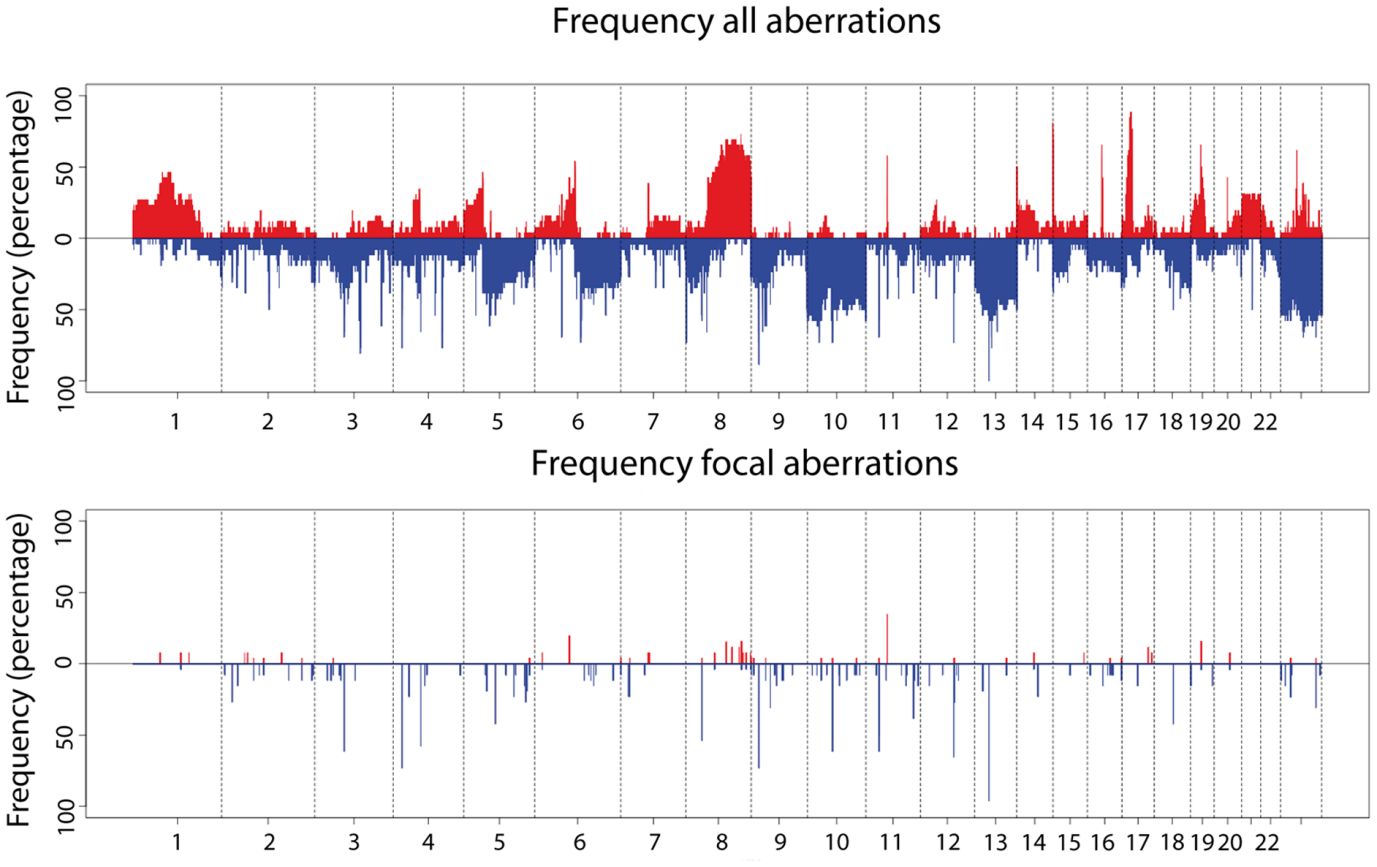
Genome-wide frequency plot of all copy number aberrations (top) and of focal copy number aberrations (bottom) in 26 osteosarcoma samples. Frequencies of gains (in red) and losses (in blue) are indicated. Vertical bars in B represent detected focal copy number aberrations (<3 Mb).

**Figure 2:**
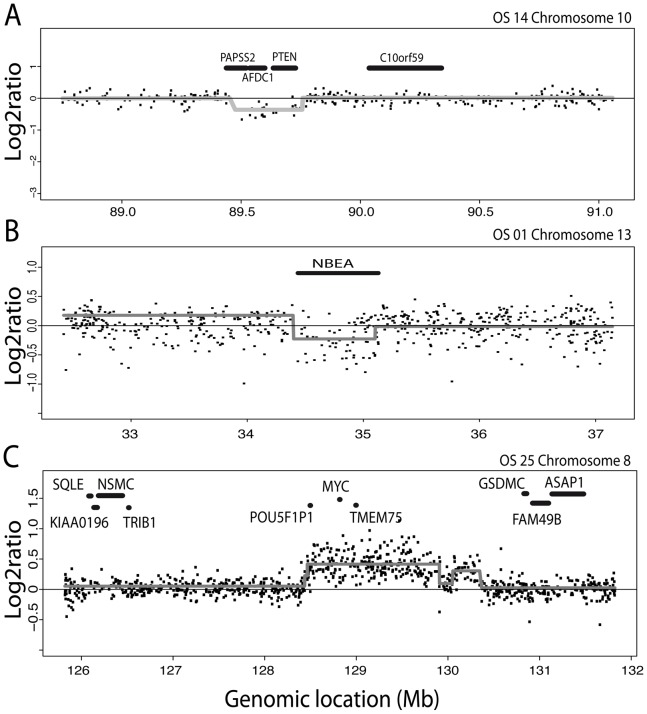
Representative examples of focal copy number aberrations. Log2ratio for each SNP is plotted against its genomic location. Gray lines indicate segment values as derived using circular binary segmentation (CBS). A, Focal deletion encompassing*PAPSS2*, *AFDC1*, and *PTEN* on chromosome 10; B, Focal deletion restricted to *NBEA* on chromosome 13; C, Focal gain including *POU5F1P1*, *MYC*, and *TMEM75* on chromosome 8.

DNA copy number profiling of 26 osteosarcoma samples showed many regions of frequent gains and losses. As shown in Fig. 1 (top panel), most frequent copy number changes were gains of chromosome arms 1p, 5p, 6p, 8q, 17p, 19 and 21 and losses of chromosome arms 3q, 5q, 6q, 8p, 9p, 10 and 13. Focal copy number changes were found in most samples with a median number of 26 (range: 4–61) for losses and 6 (range: 0–19) for gains per sample. We observed a total of 550 recurrent (n≥2) copy number changes. Representative examples of focal copy number changes are shown in Fig. 2. Out of the 550 recurrent copy number changes 266 were disregarded since they overlapped with known CNVs. Of the remaining 284 recurrent copy number changes a further 145 regions were disregarded as CNVs since both recurrent (≥2) gains and losses were detected in the dataset at the same location. This leaves a total of 139 unique genomic locations (Fig. 1, bottom panel) recognized as bonafide focal CNAs. Of these, 72 had at least one gene located within or overlapping the high frequency region (HFR), with a total of 94 genes (Table 2). These focal CNAs were significantly enriched for genes described in the Cancer Gene Census list (p-value  = 0.002, http://www.sanger.ac.uk/genetics/CGP/) and include *CBFA2T3*, *EBF1*, *FHIT*, *MYC*, *PTEN* and *RANBP17*. The focal CNAs we identified did not contain known microRNAs.

**Table 2:** High frequency regions (HFRs) of focal aberrations and gene(s) within or overlapping these HFRs.

	HFR^1^ Focal abberration			Focal^2^		Total^3^		
Chrom.	Start (bp)	End (bp)	Size (kb)	Gain	Loss	Gain	Loss	Genes
1	68394382	68461186	67	2	0	10	0	GPR177
1	166259174	166263398	4	2	0	8	0	IQWD1
1	145961855	146262362	301	2	1	8	1	FAM108A2, NBPF11
2	51022554	51022614	0.06	2	0	3	1	NRXN1
2	153790092	154905667	1116	2	0	3	1	RPRM, GALNT13
2	212844992	212863839	19	1	3	1	5	ERBB4
2	237017756	237067914	50	0	3	1	8	IQCA1
3	38177003	38246945	70	1	2	1	6	OXSR1
3	57426028	57504090	78	0	2	0	8	DNAH12L
3	59855182	59891414	36	0	2	0	9	**FHIT**
3	60208127	60420353	212	0	2	0	9	**FHIT**
3	24260772	24290280	30	0	3	0	5	THRB
3	32341229	32364439	23	0	3	0	6	CMTM8
4	90072621	90081252	9	0	2	1	3	FAM13A1
4	183476755	184028352	552	0	2	1	7	ODZ3
4	73484965	73485361	0.396	0	15	0	17	ADAMTS3
5	170214294	170645196	431	1	2	1	5	**RANBP17**
5	55470977	55502030	31	0	2	1	10	ANKRD55
5	110611609	110683366	72	0	2	0	8	CAMK4
5	135294758	135330960	36	0	2	0	8	FBXL21, LECT2
5	158336707	158375568	39	0	4	0	7	**EBF1**
5	58765648	58766102	0.454	0	5	0	12	PDE4D
5	80023854	80024518	0.664	0	11	0	14	MSH3
6	44998738	45119343	121	5	0	11	0	SUPT3H
6	13965760	14255124	289	2	1	4	1	CD83, RNF182
6	14051934	14103151	51	2	1	4	1	RNF182
6	86262042	86335470	73	0	3	0	11	SNX14, NT5E
6	110510873	110539046	28	0	4	0	12	WASF1
7	57480106	57659360	179	2	0	3	1	ZNF716
7	63149422	63486197	337	2	0	3	1	ZNF679, ZNF735
7	943615	954205	11	1	2	1	3	ADAP1
7	15536047	15536441	0.394	0	6	0	7	TMEM195
8	128437695	130578879	2141	4	1	14	1	TMEM75, **MYC**, POU5F1P1
8	124855221	124894704	39	3	0	17	0	FAM91A1
8	132030642	132421393	391	2	0	16	0	ADCY8
8	64257961	64390486	133	2	1	12	1	YTHDF3
8	145064850	145118710	54	2	1	11	1	PLEC1
8	25130171	25130291	0.12	1	14	1	15	DOCK5
9	3339487	3417226	78	1	2	1	8	RFX3
9	71289871	71308842	19	0	2	0	5	APBA1
9	87357661	87432833	75	0	3	0	5	AGTPBP1
9	33230225	33242111	12	0	8	0	12	SPINK4
10	115580402	115675237	95	1	2	1	11	NHLRC2, DCLRE1A
10	25604754	25659341	55	1	3	1	15	GPR158
10	15898437	15929438	31	0	2	0	16	C10orf97
10	38082301	38147982	66	0	2	1	11	ZNF248
10	71648150	71656573	8	0	2	0	12	PPA1
10	123566469	123752493	186	0	2	1	13	TACC2, NSMCE4A, ATE1
10	89716166	89756083	40	0	3	0	14	**PTEN**
10	72166137	72172267	6	0	4	0	12	ADAMTS14
11	20922810	20971401	49	0	2	0	3	NELL1
11	92517181	92571937	55	0	2	0	5	SLC36A4
11	104441220	104484303	43	0	2	0	4	CARD17
11	47391562	47453463	62	0	3	0	3	CUGBP1, RAPSN, PSMC3, SLC39A13
11	102486510	102663548	177	0	4	0	5	DYNC2H1
12	51710169	51719305	9	0	2	0	5	EIF4B
12	91645948	91679726	34	0	2	1	5	PLEKHG7
12	18413696	18414167	0.471	0	4	1	7	PIK3C2G
13	92502207	93946439	1444	1	2	1	12	DCT, GPC6
13	34399863	34517051	117	0	5	0	14	NBEA
14	59138607	59161895	23	2	1	5	1	RTN1
14	69961223	69998909	38	0	6	0	6	ADAM21, ADAM21P
15	59943982	60046989	103	0	2	1	3	VPS13C
16	87557619	87557679	0.06	1	2	1	8	**CBFA2T3**
16	87527215	87549395	22	1	3	1	9	**CBFA2T3**
16	71734726	71798103	63	0	2	0	6	C16orf47
16	45728834	45887805	159	0	4	0	6	ITFG1, NETO2
17	37018570	37057586	39	0	4	1	7	KRT42P, KRT17, KRT16
19	33996261	34984966	989	4	1	12	1	C19orf12, PLEKHF1, POP4, VSTM2B, UQCRFS1, UQCRFSL1
19	532070	556653	25	0	4	1	4	HCN2, BSG
19	60711906	60737394	25	0	4	1	6	SBK2
X	33147645	33243866	96	1	6	1	15	DMD

1 HFR: common region of focal aberration (gain or loss);

2 Focal: number of samples with focal aberration;

3 Total: total number of samples containing the HFR of the focal aberration. Genes in bold have been reported in the Cancer Gene Census list.

### Expression of genes in focal CNAs

**Figure 3:**
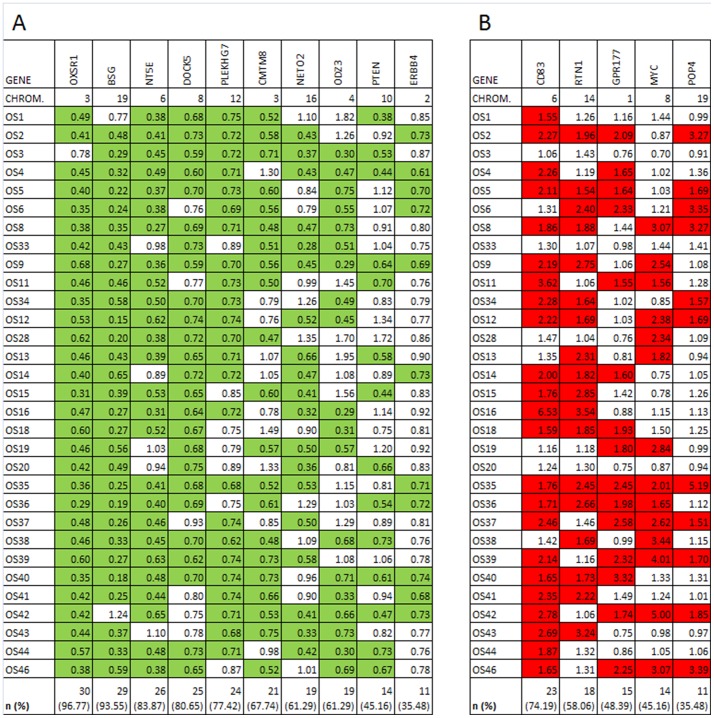
Genes in focal losses with underexpression (FC<0.75, A) and in focal gains with overexpression (FC>1.5, B) in more than 35% of osteosarcomas. Green: expression fold change (FC) <0.75 of the gene in the tumor relative to osteoblasts (underexpression). Red: expression fold change (FC) >1.50 of the gene in the tumor relative to osteoblasts (overexpression). For each gene, the number (n) and fraction (%) of osteosarcomas with the indicated fold change (FC) are given.

Whole genome expression data was available for 31 osteosarcoma samples, including 20 samples from the SNP-array analysis set. These data were used for further analysis of our candidate list of potential driver genes (Table 2). For 85 of the 94 genes at least 1 probe was available on the expression array. Expression fold changes for these genes were calculated by normalizing the intensity of the average signal in the tumor sample against the average signal in the reference osteoblast sample. The genes in the focal copy number losses and in the focal copy number gains were separately assayed for underexpression (expression fold change ≤0.75) and overexpression (expression fold change ≥1.5), respectively. All data are shown in S1 Table. From this table a top candidate gene list was extracted with aberrant expression (underexpression in case of focal loss, overexpression in case of focal gain) in ≥35% of the tumors (Fig. 3). Besides the known tumor suppressor genes *DOCK5* and *PTEN*, genes *OXSR1*, *BSG*, *NT5E*, *PLEKHG7*,*CMTM8*, *NETO2*, *ODZ3*, and *ERBB4* adhered to our criteria and were qualified as novel candidate tumor suppressor genes. Besides the known oncogene *MYC*, genes *CD83*, *RTN1*, *GRP177*, and *POP4* adhered to our criteria and were qualified as novel candidate oncogenes.

### Validation analysis

To confirm our findings of the proposed candidate genes in an independent dataset we used expression data for 84 osteosarcoma samples and 3 osteoblast samples, which were generated by Kuijjer et al [15]. Expression fold changes for our candidate genes were calculated by normalizing their expression level in the osteosarcoma samples against the mean expression level in the osteoblast reference samples. Next, they were assayed for their frequency of underexpression (expression fold change ≤0.75, in case of a candidate tumor suppressor gene) or overexpression (expression fold change ≥1.5, in case of a candidate oncogene) in the osteosarcoma samples (see S1 Fig.). In Table 3, the frequencies of involvement of our candidate driver genes in this validation set are summarized and compared with those in our own osteosarcoma set. Considering a frequency of involvement for a driver gene of at least 35% as a convincing event, we conclude that the validation set confirms our identification of *OXSR1, BSG, NT5E, CMTM8*, and *NETO2* as novel candidate tumor suppressor genes and of *CD83, RTN1*, and *GPR177* as novel oncogenes.

**Table 3:** Validation of identified candidate tumor suppressor genes and oncogenes in an independent dataset.

Gene	Our set	Validation set
Candidate tumor suppressor gene		
OXSR1	97	89
BSG	94	70
NT5E	84	93
(DOCK5	81	90)
PLEKHG7	77	0
CMTM8	68	65
NETO2	61	60
ODZ3	61	NI^1^
(PTEN	45	NI^2^)
ERBB4	35	0
Candidate oncogene		
CD83	74	51
RTN1	58	74
GPR177	48	43
(MYC	45	4)
POP4	35	18

Frequency of involvement (percentage underexpression of a candidate tumorsuppressor gene and percentage overexpression of a candidate oncogene) in the original and validation set

Known tumor suppressor genes (*DOCK5*, *PTEN*) and oncogene (*MYC*) between brackets

1 No information. Applied probe does not recognize *ODZ3* in 4q35.1

2 No information. Applied probe recognizes not only *PTEN* on 10q23.31, but also the expressed pseudogene (*PTENP1*) in 9p13.3.

## Discussion

Due to the complex landscape of genomic rearrangements in osteosarcomas the identification of driver genes remains a challenging task. Nonetheless, by analysis of focal aberrations, we identified potential new and known genes driving osteosarcoma tumorigenesis. In our set of 26 osteosarcoma samples, 94 genes were identified within recurrent focal aberrations. Some were found in 2 samples only, others in a large portion of the samples, such as *DOCK5* (14/26) and *ADAMTS3* (15/26) (Table 2). It should be noted that the previously published amplifications in the 17p11.2-p12 region and the potential oncogenes therein [16] were not identified in this study since these amplifications were generally large (>3 Mb), and hence not recognized as focal CNAs in this study.

For most of the genes in the focal aberrations (85 of 94) expression data in a set of 31 osteosarcoma samples were available. This allowed us to identify the genes of which the expression status (over- or underexpression) in these tumors was frequently (>35%) in accordance to their copy number status (gain or loss), as deduced from the focal aberration analyses. These were, in addition to the known tumor suppressor genes *PTEN* and *DOCK5* and the known oncogene *MYC*, in total 8 candidate tumor suppressor genes and 4 candidate oncogenes as potential new driver genes in osteosarcoma tumorigenesis (Table 3). As discussed below for the individual genes, validation using an independent dataset, confirmed the candidacy of most of the driver genes that we identified.

### Known tumor suppressor genes and oncogenes found in our analysis

The strength of the applied approach of focal aberration analysis might be concluded from the enrichment of the focal CNAs we identified in our osteosarcoma dataset with driver genes from the Cancer Census list [23] and in particular the identification of *PTEN*, *DOCK5*, and *MYC* as known driver genes in osteosarcoma. For instance, *PTEN* has been implicated in osteosarcoma tumorigenesis in a multitude of studies. Loss of the *PTEN* gene has been reported in patient samples [25] and lower *PTEN* expression levels in cell lines [26]. However, because the available probe in the validation set proved to be not specific to *PTEN*, we could not confirm its frequent involvement as a tumor suppressor gene in this set. We found frequent underexpression of *DOCK5* in our set as well as in the validation set. Expression of *DOCK5* is essential for bone differentiation in osteoclasts [27]. Recently, *DOCK5* expression was shown to be down-regulated in osteosarcoma [28]. The *MYC* oncogene is involved in many tumor types, including carcinomas of cervix, lung, breast, colon, and stomach, and also in osteosarcoma [29]. MYC acts as a cell proliferation controller and overexpression, as found in a multitude of tumors as well as in our dataset, drives higher proliferation and blocks cell differentiation. Furthermore, a secondary effect of MYC overexpression is the induction of chromosomal instability by faulty control of the G1-S checkpoint [30]. For unknown reasons, we could not confirm the frequent overexpression of *MYC*, as noted in our osteosarcoma set, in the validation set. Unfortunately, no expression data for another *MYC* probe in the latter set was available to substantiate this unexpected result.

### Newly identified candidate tumor suppressor genes

In our analysis eight new candidate tumor suppressor genes were identified, *OXSR1* (3p22.2), *BSG*(19p13.3), *NT5E*(6q14.3), *PLEKHG7*(12q22), *CMTM8*(3p22.3), *NETO2*(16q12.1), *ODZ3*(4q35.1) and *ERBB4*(2q34). However, since the involvement of *PLEKHG7* and *ERBB4* could not be confirmed in the validation set and conflicting data have been reported for *OXSR1*, *BSG*, *NT5E*, and *NETO2* in other tumor types (summarized in Table 4), we consider these as less probable candidate tumor suppressor genes in osteosarcoma tumorigenesis.

**Table 4:** Excluded newly identified driver genes.

Gene	Reason for exclusion
Candidate tumor suppressor gene(s)	
*PLEKHG7, ERBB4*	Underexpression in our set, but no confirmation in validation set
*OXSR1, NT5E, NETO2*	Underexpression in our set and validation set, but conflicting expression data in other tumor types [46]–[51]
*BSG*	Underexpression in our set and validation set, conflicting with oncogenic role in other tumor types [52]–[55]
Candidate oncogene	
*CD83*	Overexpression in our set and validation set of this cell surface marker most probably related to altered tumor microenvironment, but not to tumor formation [56]
*RTN1*	Overexpression in our set and validation set conflicts with tumor suppressor role in other tumor types [57]–[59]

In several tumor types, indications have been found for a tumor suppressor function of the *CMTM8* gene product. In osteosarcoma, it was demonstrated that CMTM8, also known as CKLFSF8, suppresses the EGFR signaling pathway [31]. *CMTM8* underexpression may therefore result in upregulation of EGFR signaling. The latter was recently shown to suppress osteoblast differentiation and to inhibit expression of the osteoblastic transcription factors Runx2 and Osterix, which may lead to the development of immature osteoblastic-like cells characteristic of osteosarcoma [32]. In other tumor types, overexpression of *CMTM8* has been shown to result in tumor cell apoptosis [33], [34]. In addition, in hepatocellular carcinoma cells and immortalized breast epithelial cells (MCF-10A), downregulation of *CMTM8* was found to result in a transition from an epithelial to a mesenchymal phenotype. This transition results from the loss of c-Met inhibition by CMTM8, which in turn activates migration, invasive growth and cancer malignancy [35], [36]. Interestingly, c-Met is known to be upregulated in human sarcomas [37].We found *CMTM8* underexpression in a considerable number of cases in our original set and in the validation set. This gene may therefore have a tumor suppressor gene function in osteosarcoma tumorigenesis.

We found *ODZ3* to be underexpressed in a considerable fraction of the investigated osteosarcomas but, because of lack of a specific probe, were unable to validate the expression data for this gene in the independent set. Deletion of *ODZ3* and low mRNA expression has been noted to occur in neuroblastoma [38]. In addition, in the latter study the low expression level of this gene proved to be associated with a poor prognosis. Whether *ODZ3* has a similar tumor suppressor gene function in osteosarcoma remains to be established.

### Newly identified candidate oncogenes

We identified three genes located in focal gains and overexpressed in a significant fraction of both osteosarcoma sets: *CD83* (6p23), *RTN1* (14q23.1), and *GPR177* (*WLS*/*EVI*) (1p31.3). However, as briefly explained in Table 4, we consider *CD83* and *RTN1* as less probable candidate oncogenes in osteosarcoma tumorigenesis.

No information is available about the expression of *GPr177(WLS/EVI)* in osteosarcomas. It is known that physiologically normal GPR177 protein levels are essential for proper osteoblast differentiation and mineralization [39]. The *GPR177* gene encodes an upstream regulator of the Wnt signaling pathway. However, the status of this pathway in osteosarcoma is unclear. Some papers report active Wnt signaling [40], [41], while others find that the Wnt signaling pathway is inactivated in this tumor [42], [43]. GPR177 has been found to be overexpressed in glioma [44]. Moreover, GPR177 proved to be indispensable for Wnt-induced breast tumor formation [45]. In accordance with the latter observations, we found frequent overexpression of *GPR177* in both osteosarcoma sets. Taken together, these data suggest a potential oncogenic role for *GPR177* in osteosarcoma tumorigenesis.

## Conclusions

Based on their frequent aberrant copy number in our osteosarcoma set, the frequent under-, respectively, overexpression in our osteosarcoma set as well as in a validation set, and their documented involvement in other tumor types, we identified *CMTM8*, and possibly also *ODZ3*, as a new candidate tumor suppressor gene, and *GPR177* as a new candidate oncogene involved in osteosarcoma tumorigenesis.

## Supporting Information

S1 Fig Genes in focal losses with underexpression (FC<0.75, A) and in focal gains with overexpression (FC>1.5, B) in more than 35% of osteosarcomas in the validation dataset. Green: expression fold change (FC) <0.75 of the gene in the tumor relative to osteoblasts (underexpression). Red: expression fold change (FC) >1.50 of the gene in the tumor relative to osteoblasts (overexpression). For each gene, the number (n) and fraction (%) of osteosarcomas with the indicated fold change (FC) are given.
(XLSX)

S1 Table Expression fold change for all genes in focal losses (upper panel) or focal gains (lower panel) in 31 osteosarcoma samples. Green: expression fold change (FC) <0.75 of the gene in the tumor relative to osteoblasts (underexpression). Red: expression fold change (FC) >1.50 of the gene in the tumor relative to osteoblasts (overexpression). For each gene, the number (n) and fraction (%) of osteosarcomas with the indicated fold change (FC) are given.
(XLSX)
